# A tomato HD-zip I transcription factor, VAHOX1, acts as a negative regulator of fruit ripening

**DOI:** 10.1093/hr/uhac236

**Published:** 2022-10-19

**Authors:** Fenfen Li, Mengjie Fu, Shengen Zhou, Qiaoli Xie, Guoping Chen, Xuqing Chen, Zongli Hu

**Affiliations:** Laboratory of Molecular Biology of Tomato, Bioengineering College, Chongqing University, Chongqing, China; Laboratory of Molecular Biology of Tomato, Bioengineering College, Chongqing University, Chongqing, China; Laboratory of Molecular Biology of Tomato, Bioengineering College, Chongqing University, Chongqing, China; Laboratory of Molecular Biology of Tomato, Bioengineering College, Chongqing University, Chongqing, China; Laboratory of Molecular Biology of Tomato, Bioengineering College, Chongqing University, Chongqing, China; Institute of Grassland, Flowers and Ecology, Beijing Academy of Agriculture and Forestry Sciences, Beijing, China; Laboratory of Molecular Biology of Tomato, Bioengineering College, Chongqing University, Chongqing, China

## Abstract

Homeodomain-leucine zipper (HD-Zip) transcription factors are only present in higher plants and are involved in plant development and stress responses. However, our understanding of their participation in the fruit ripening of economical plants, such as tomato (*Solanum lycopersicum*), remains largely unclear. Here, we report that *VAHOX1*, a member of the tomato HD-Zip I subfamily, was expressed in all tissues, was highly expressed in breaker+4 fruits, and could be induced by ethylene. RNAi repression of *VAHOX1* (*VAHOX1-*RNAi) resulted in accelerated fruit ripening, enhanced sensitivity to ethylene, and increased total carotenoid content and ethylene production. Conversely, *VAHOX1* overexpression (*VAHOX1-*OE) in tomato had the opposite effect. RNA-Seq results showed that altering *VAHOX1* expression affected the transcript accumulation of a series of genes involved in ethylene biosynthesis and signal transduction and cell wall modification. Additionally, a dual-luciferase reporter assay, histochemical analysis of GUS activity and a yeast one-hybrid (Y1H) assay revealed that VAHOX1 could activate the expression of *AP2a*. Our findings may expand our knowledge about the physiological functions of HD-Zip transcription factors in tomato and highlight the diversities of transcriptional regulation during the fruit ripening process.

## Introduction

The ripening process of fleshy fruits involves complicated physiological and biochemical characteristics, which are linked to dramatic changes in colour, flavour, aroma, texture, and nutritional content [1]. Fleshy fruits are divided into climacteric or nonclimacteric fruits based on whether ethylene production and respiration increase during ripening [2, 3]. Climacteric fruits, including tomato, apple, banana, peach, and most stone fruits, experience dramatic induction of respiration and an increase in autocatalytic ethylene production during ripening initiation; however, these characteristic bursts are non-existent in non-climacteric fruits, such as citrus, strawberry, and grape [4, 5].

Fruit ripening is coordinated by endogenous hormones, environmental signals, and complex genetic regulators [6]. Ethylene is the major trigger of climacteric fruit ripening and blocking ethylene biosynthesis or signal transduction can effectively inhibit ripening initiation [7–9]. In addition to ethylene, transcription factors (TFs) are also crucial for the ripening of climacteric fruits. Therefore, it is of great significance to investigate the functions of TFs to improve the complex regulatory network of fruit ripening. A breakthrough regarding ripening regulation is based on the characteristics of several well-known genetic mutants, such as *ripening inhibitor* (*rin*), *Colorless non-ripening* (*Cnr*), and *nonripening* (*nor*) [10]. The loci of these tomato ripening-related mutants all harbour transcription factors. *RIN*, *CNR*, and *NOR* encode MADS-box protein, SBP-box protein, and NAC domain protein, respectively, and modulate the transcription of ethylene and carotenoid biosynthesis pathway-related genes [1, 4, 11–13]. An increasing number of studies have identified other crucial ripening-associated transcription factors and provided deeper insights into the molecular basis of fruit ripening. Auxin response factor MdARF5 regulates apple fruit ripening by activating ethylene biosynthetic genes [14]. In peach, PpIAA1 and PpERF4 function as a complex to enhance the mRNA accumulation of fruit ripening-associated genes [15]. The MADS-box TF MaMADS36 is critical for banana fruit ripening [16]. FUL1 and FUL2, two MADS-box family members, are characterized as ethylene-independent regulators that participate in tomato fruit ripening [17, 18]. The tomato GRAS transcription factor SlGRAS4 can directly activate ethylene biosynthesis genes (*SlACO1* and *SlACO3*) and repress *SlMADS1* to accelerate fruit ripening [19]. MYB70 controls tomato fruit ripening by directly repressing ethylene biosynthesis genes [20]. Downregulation of *APETALA2a* (*AP2a*)/*SlAP2a* leads to early fruit ripening and increased ethylene production [21, 22].

The HD-Zip TFs are found only in plants. HD-Zip proteins include a homeodomain (HD) and a homeobox domain-associated leucine zipper (Zip) motif [23], which have been primarily divided into four subfamilies named I to IV on the basis of their structural and functional characterizations [24]. Previous studies point out that HD-Zip TFs are implicated in plant growth, development and stress responses. For example, AtHB1 functions downstream of PIF1 to promote hypocotyl growth and regulates genes related to cell elongation in Arabidopsis [25]. Arabidopsis plants with enhanced expression of *GmHDZ20* display defects in leaf morphology, silique length, and seed number [26]. *Arabidopsis thaliana ATHB12* boosts leaf growth by controlling cell expansion and endoreduplication [27]. Recently, a study revealed that SlHD8 physically interacts with SlJAZ4 to mediate JA-induced trichome elongation in tomato [28]. *Oshox22* negatively modulates salt and drought tolerance in rice [29]. Tomato SlHZ24 modulates AsA biosynthesis [30]. Tomato serves as an ideal model species for research on fleshy fruit development and ripening [10, 31, 32]. Nevertheless, few reports have examined the participation of HD-Zip TFs in regulating tomato fruit ripening, with the exception of one study that reports that the tomato HD-Zip gene *LeHB-1* is involved in controlling fruit ripening and floral organogenesis [33]. The *LeHB-1* gene is more highly expressed in developing fruits, whereas its mRNA levels decrease at the onset of ripening. Virus-induced gene silencing of *LeHB-1* reduces *ACO1* mRNA levels and delays tomato fruit ripening. Conversely, ectopic overexpression of *LeHB-1* alters floral organ morphology [33].

To investigate the roles of HD-Zip TFs in fruit ripening, *VAHOX1* was chosen for our work because it is the tomato gene most closely related to *LeHB-1*, and *VAHOX1* shows the highest transcript abundance in fruit compared with other tomato tissues in an online analysis in the Tomato eFP Browser (http://bar.utoronto.ca/efp_tomato/cgi-bin/efpWeb) (Fig. S1, see online supplementary material). Here, we found that the HD-Zip I subfamily gene *VAHOX1* exhibited the highest transcription level in breaker+4 fruits and played a negative regulatory role in tomato fruit ripening. RNAi repression of *VAHOX1* led to premature fruit ripening, enhanced ethylene sensitivity, and increased total carotenoid accumulation. Conversely, the upregulation of *VAHOX1* delayed ripening initiation, weakened the sensitivity to ethylene and reduced the total carotenoid content. Further study showed that VAHOX1 could positively regulate the promoter activity of *AP2a*, suggesting that VAHOX1 modulates the mRNA level of *AP2a* to affect fruit ripening. These findings demonstrate the involvement of HD-Zip TF in regulating climacteric fruit ripening.

## Results

### Features of tomato transcription factor VAHOX1

**Figure 1:**
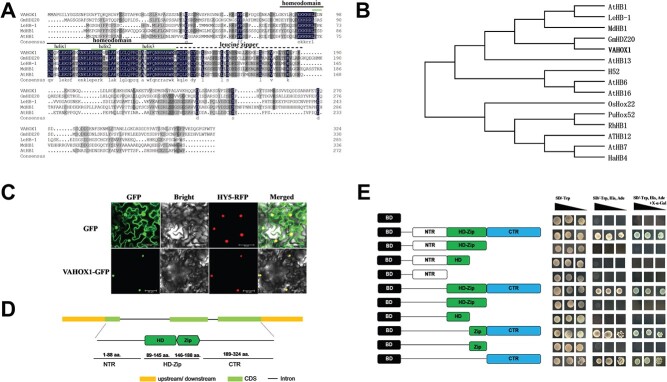
Sequence analysis, subcellular localization and transactivation activity of tomato *VAHOX1*. **a** Amino acid alignment of VAHOX1 and GmHDZ20, MdHB1, LeHB-1, and AtHB1 proteins. **b** Phylogenetic analysis of VAHOX1 and class-I HD-Zip proteins from Arabidopsis, MdHB1 from apple, GmHDZ20 from soybean, HaHB-4 from sunflower, OsHox22 from rice, PuHox52 from poplar, RhHB1 from rose, LeHB-1 and H52 from tomato. **c** Subcellular localization of VAHOX1. The control GFP signal was present throughout the cells, whereas VAHOX1-GFP was restricted to the nucleus. HY5-RFP was used as a nuclear maker. Three independent experiments were performed. Bars, 50 μm. **d** Genomic structure and conserved domains of VAHOX1. aa.: amino acid; CDS, coding sequence; CTR: C-terminus; HD-Zip: homeodomain-leucine zipper; NTR: N-terminus. **e** Transcriptional activation activity of the different parts of VAHOX1 in yeast. The experiment was repeated three times. BD: empty pGBKT7 vector.

*VAHOX1* gene contains three exons and two introns and encodes a 324-amino-acid (aa) protein with the conserved HD and Zip domains (Fig. 1a, d). Phylogenetic analysis indicated that VAHOX1 was grouped with other plant HD-Zip I proteins, GmHDZ20 (similarity of 44.87%) from soybean (*Glycine max*), MdHB1 (similarity of 31.81) from apple (*Malus domestica*), LeHB-1 (similarity of 30.91%) from tomato (*S. lycopersicum*) and AtHB1 (similarity of 29.94%) from Arabidopsis (*A. thaliana*), respectively (Fig. 1b).

To investigate the subcellular localization of VAHOX1, a *35S::VAHOX1*-*GFP* fusion vector was created and transiently expressed in tobacco leaves. We found that the control GFP signal was present throughout the cells, whereas VAHOX1 was restricted to the nucleus, suggesting that VAHOX1 may function in the nucleus (Fig. 1c).

Subsequently, to determine whether VAHOX1 possesses a transcriptional activation function and where its transactivation site is located, the full-length and various truncated forms of *VAHOX1* were expressed by transforming the recombinant BD vectors into yeast strain Y2H-Gold (Fig. 1d, e). Vigorous growth of yeast transformed with each recombinant plasmid was observed on the SD-Trp plate. However, only the transformants with pGBKT7-VAHOX1 constructs that include the C-terminal region (CTR, 189–324 aa) grew well on SD-Trp/His/Ade medium (Fig. 1e). These results suggest that VAHOX1 possesses a transcriptional activation function and that the C-terminus of VAHOX1 is critical to its transcriptional activation activity in yeast cells.

### Expression analysis of *VAHOX1*

**Figure 2:**
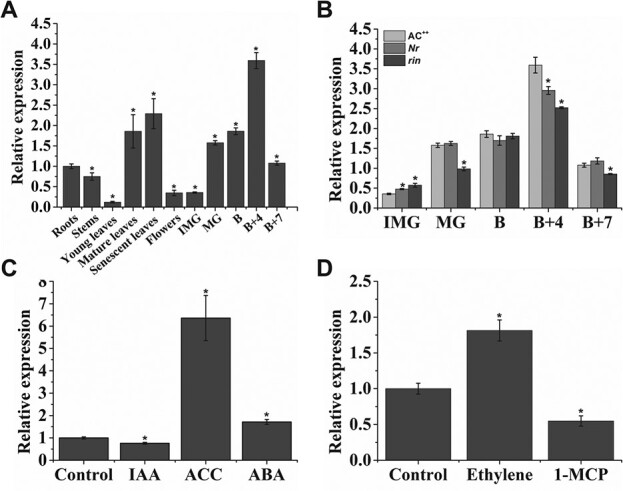
Expression patterns of *VAHOX1.***a** Relative transcript levels of *VAHOX1* in the different wild-type tomato tissues. The transcript accumulation of *VAHOX1* in roots was normalized to 1. **b** Transcript levels of *VAHOX1* in the wild-type and mutant fruits at the different stages of ripening. **c** and **d** Response of *VAHOX1* to several ripening-related hormones, including IAA, ABA, ACC, ethylene, and the ethylene inhibitor 1-MCP. Values are means ± standard error (SE) based on three biological replicates. Asterisks indicate significant differences (*P* < 0.05). The asterisks in the following text indicate the same as here.

To reveal the physiological significance of *VAHOX1*, we first performed qRT–PCR experiments to examine the transcript accumulation of *VAHOX1* in various tomato tissues. Remarkably, *VAHOX1* was expressed in all tissues examined, and during fruit development, the *VAHOX1* transcript rapidly increased at first and then peaked at the B + 4 stage, after which it declined at the B + 7 stage (Fig. 2a). This result indicates that *VAHOX1* may act in regulating fruit ripening. Additionally, we observed a similar *VAHOX1* expression trend in the fruits of the *Nr* and *rin* mutants, suggesting that the expression of *VAHOX1* may be not associated with the single-locus *RIN* or *Nr* (Fig. 2b). Numerous studies describe the importance of phytohormones, such as ethylene, abscisic acid [45–47] and auxin [48–50], during fruit ripening. To investigate whether the expression of *VAHOX1* is regulated by phytohormones, we next assessed the transcript accumulation of *VAHOX1* in the MG fruits of the WT plants treated with IAA, the ethylene precursor ACC, ABA, ethylene and the ethylene inhibitor 1-MCP. *VAHOX1* was greatly induced by ACC and ethylene but repressed by 1-MCP, indicating that *VAHOX1* may respond to ethylene (Fig. 2c, d).

### Effect of *VAHOX1* on fruit ripening

To better understand the function of *VAHOX1* in tomato, four independent *VAHOX1*-RNAi and three *VAHOX1*-OE transgenic lines were obtained (data not shown). The expression of *VAHOX1* in fruits at B, B + 4 and B + 7 stages was confirmed by qRT–PCR analysis. The transcript accumulation of *VAHOX1* was significantly increased and reduced in the *VAHOX1*-OE and *VAHOX1*-RNAi fruits, respectively (Fig. S2A, see online supplementary material). The expression of *LeHB-1*, the close homolog of *VAHOX1*, in the *VAHOX1*-RNAi lines was not altered (Fig. S2B, see online supplementary material). Two RNAi (RNAi5 and RNAi9) and two OE (OE25 and OE33) independent lines were selected for detailed study.

**Figure 3:**
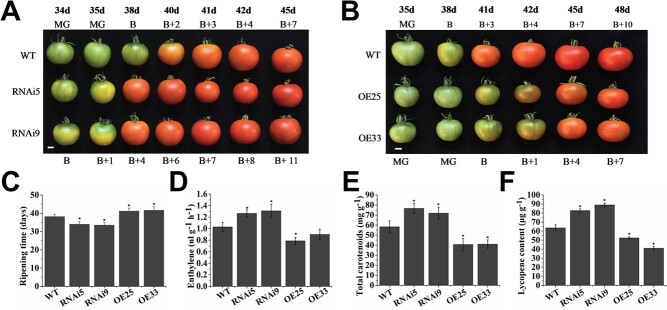
VAHOX1 affects fruit ripening in tomato. Fruits from *VAHOX1*-RNAi (**a**) and *VAHOX1*-OE (**b**) lines show earlier and delayed ripening phenotypes compared with the control plants, respectively. d: days. Bars, 1 cm. **c** Average days from anthesis to B stage in the WT, *VAHOX1*-RNAi, *VAHOX1*-OE fruits. **d** Comparison of ethylene concentrations in the control, RNAi, and OE fruits. Values represent the means based on at least six individual fruits. Comparison of total carotenoid (**e**) and lycopene (**f**) accumulation in the control and transgenic fruits at the B + 4 stage. Error bars indicate the SE.

A marked alteration in fruit ripening was observed. Relative to the wild-type fruits, the *VAHOX1*-RNAi fruits displayed earlier ripening, whereas the *VAHOX1*-OE fruits exhibited delayed ripening. At 38 days post-anthesis, the control fruits were in the B stage, whereas the *VAHOX1*-OE fruits were still in the MG stage, and the *VAHOX1*-RNAi fruits had reached the red stage (Fig. 3a, b). On average, the breaking time of *VAHOX1-*RNAi plants was 4 days earlier than that of wild-type fruits, but the OE lines exhibited a 3-day delay (Fig. 3c). Given the importance of ethylene for fruit ripening, the ethylene production of *VAHOX1*-silenced and *VAHOX1*-OE fruits at the B + 4 stage was investigated. Compared with WT, the level of ethylene production was increased and reduced in the *VAHOX1*-silenced and *VAHOX1*-OE fruits, respectively, suggesting that VAHOX1 may have a negative regulatory function in ethylene production (Fig. 3d). To characterize the changes in fruit colour of transgenic lines and wild-type plants, the pigment accumulation was measured. We found that the amounts of total carotenoids and lycopene in the *VAHOX1-*silenced fruits were higher than those in the control fruits, whereas those in the *VAHOX1-*OE fruits were lower (Fig. 3e, f). These results suggest that VAHOX1 may function as an inhibitor to regulate fruit ripening.

### VAHOX1 affects ethylene sensitivity in tomato

**Figure 4:**
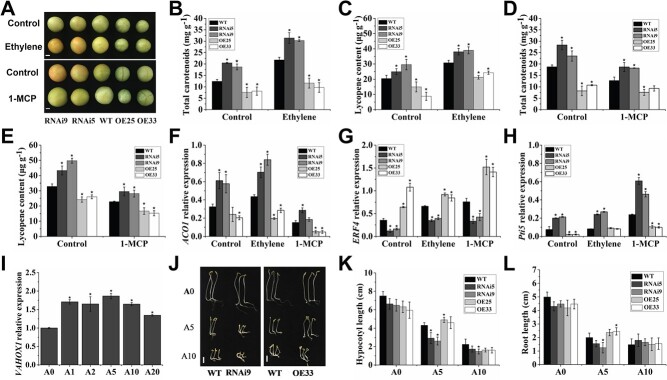
Response of tomato plants to ethylene. **a** Phenotype of the MG fruits treated with ethylene and 1-MCP for 2 days and 4 days, respectively. Bars, 1 cm. **b**–**e** show the accumulation of total carotenoids and lycopene in the wild-type and transgenic fruits treated with ethylene and 1-MCP, respectively. The transcript levels of *ACO1* (**f**), *ERF4* (**g**), and *Pti5* (**h**) in the WT and transgenic fruits treated with ethylene and 1-MCP for 2 days. **i** Accumulation of *VAHOX1* transcripts in the WT seedlings treated with air (A0), 1.0 (A1), 2.0 (A2), 5.0 (A5), 10.0 (A10), and 20.0 (A20) μM, respectively. The accumulation in the seedlings treated with air is standardized to 1.0. In (**b**) to (**i**), error bars indicate the SE based on three replicates. **j** Phenotypes of the transgenic seedlings treated with ACC. Bars, 1 cm. Hypocotyl (**k**) and root (**l**) lengths of seedlings under air (A0), 5 μM ACC (A5), and 10 μM ACC (A10), respectively. Values are means ± SE (*n* ≥ 16) of three replicates.

We further investigated the connection between *VAHOX1*-OE/RNAi and ethylene in fruits. The detached MG fruits of the control and transgenic plants were treated with air, ethylene, and 1-MCP (Fig. 4a). External ethylene accelerated colour change in the *VAHOX1-*RNAi fruits after treatment for 2 days, and the RNAi fruits became faintly orange-red, whereas the WT fruits were orange. Additionally, RNAi fruits exhibited colour changes earlier than the wild-type fruits after 1-MCP treatment. After 4 days of 1-MCP treatment, the RNAi fruits were light orange, but the WT fruits were still at the MG stage and displayed no notable colour change. Conversely, there seemed to be no obvious colour change after the same treatments of *VAHOX1-*OE fruits (Fig. 4a). After treatments with ethylene or 1-MCP, the contents of total carotenoids and lycopene were increased in *VAHOX1-*silenced fruits compared with the control fruits, but in *VAHOX1-*OE fruits, the opposite results were observed (Fig. 4b–e). Moreover, the ethylene biosynthesis gene *ACO1* and signalling gene *Pti5*/*ERF.C6* was induced in *VAHOX1*-RNAi fruits but repressed in OE fruits (Fig. 4f, h), and the ethylene signalling gene *ERF4* showed decreased expression in the RNAi fruits but increased expression in the *VAHOX1*-OE fruits relative to the WT after treatments (Fig. 4g). Our data suggest that silencing *VAHOX1* enhanced the response of fruits to ethylene in tomato plants, while the overexpression of this gene weakened the response of the fruits.

Meanwhile, we found that the expression of *VAHOX1* was upregulated in the WT seedlings treated with ACC (Fig. 4i). This observation motivated further investigation of ethylene sensitivity in the nonfruit tissues of transgenic plants by an ethylene triple-response experiment (Fig. 4j). There was no obvious difference in root and hypocotyl elongation between nontransgenic and transgenic seedlings in the absence of ACC. However, the lengths of hypocotyls and roots were reduced after ACC treatment (5 μM). Compared with the control wild-type seedlings, *VAHOX1-*OE seedlings displayed reduced inhibition of root growth and hypocotyl elongation. In contrast, the inhibition was enhanced in RNAi seedlings, in line with the ethylene sensitivity in fruits (Fig. 4j–l). In addition, the accumulation of *VAHOX1* transcripts in *VAHOX1*-RNAi and *VAHOX1*-OE seedlings were lower and higher than those in wild-type seedlings, respectively, with or without ACC treatment (Fig. S3, see online supplementary material), which confirmed the ethylene triple response presented in the seedlings of *VAHOX1*-silenced and *VAHOX1*-OE lines.

### VAHOX1 affects fruit softening

**Figure 5:**
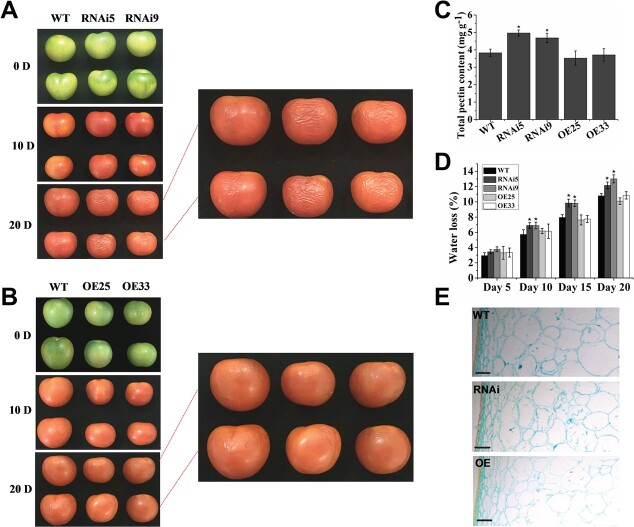
VAHOX1 affects tomato shelf life. Comparison of WT, *VAHOX1*-RNAi (**a**) and *VAHOX1*-OE (**b**) fruits 10 and 20 days after harvesting at B stage. **c** Assessment of total pectin content in the control and transgenic fruits. Each data represents a mean of three replicates. **d** Comparison of water loss rate of control and transgenic tomato fruits. Tomato fruits were harvested at the B stage, and then weighted every 5 days during the 20 days of storage. Values are means ± SE (*n* = 15). **e** Microscopic observation of the control and transgenic fruits stored for 20 days. Bars, 50 μm.

Softening is a considerable characteristic of fleshy fruit ripening. Pectin is one of the key components of the plant cell wall, and its content and proportions can affect fruit firmness and softening. The *VAHOX1-*RNAi fruits exhibited higher total pectin content than wild-type fruits (Fig. 5c). Given the effect of *VAHOX1* on tomato fruit ripening, a storage test was performed to observe the shelf life of the *VAHOX1*-OE/RNAi fruits. The *VAHOX1-*RNAi fruits exhibited a higher water loss rate and more wrinkles than the WT and OE fruits during postharvest storage. The *VAHOX1-*RNAi fruits wrinkled after 10 days of storage, while the *VAHOX1*-OE and WT fruits displayed similar symptoms up to 20 days postharvest (Fig. 5a, b and d). Anatomical analysis showed that the pericarp cell shape of the WT and OE fruits was rounder and more regular than that of *VAHOX1-*RNAi fruits (Fig. 5e). Expression of cell wall modification-related genes *TBG4*, *TBG7*, *XTH9*, *HEX*, and *PE* were detected in the WT and transgenic fruits stored for 20 days. Relative to the WT, the expression of *TBG4*, *TBG7*, and *HEX* was elevated in the *VAHOX1-*RNAi fruits. *TBG4* and *HEX* were reduced in the *VAHOX1*-OE fruits, but *TBG7* was not (Fig. S4a, b and d, see online supplementary material). *XTH9* expression was decreased in the *VAHOX1-*RNAi fruits but enhanced in the *VAHOX1*-OE fruits (Fig. S4c, see online supplementary material). The mRNA levels of *PE1* were lower in the RNAi and OE fruits than in the wild-type fruits (Fig. S4e, see online supplementary material).

### The expression of ripening-associated genes is altered in *VAHOX1* transgenic fruits

**Figure 6:**
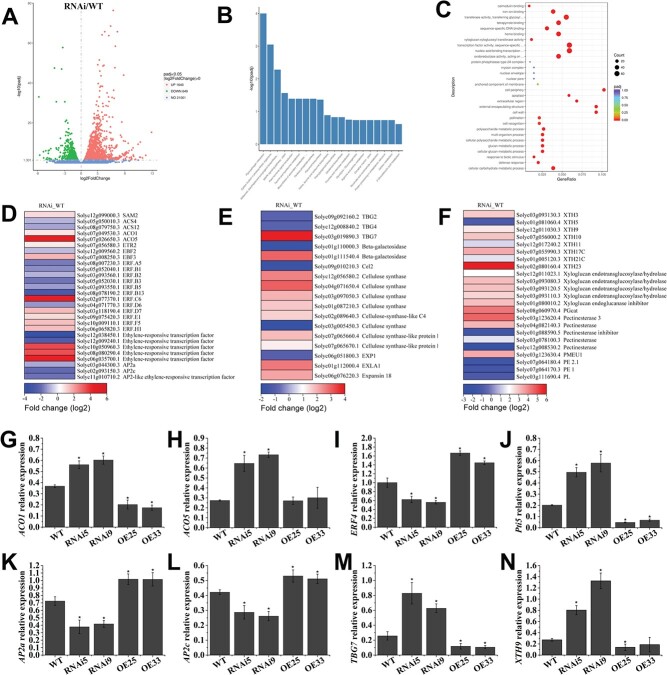
RNA-seq analysis of *VAHOX1*-RNAi fruits. **a** RNA-seq data visualized by volcano plots. Red and green points show upregulated and downregulated genes in the *VAHOX1*-RNAi fruits versus the WT fruits (RNAi_WT), respectively. Blue points represent genes with no significant difference. KEGG pathway enrichment analysis (**b**) and GO classification (**c**) of genes differentially expressed in the *VAHOX1*-RNAi and WT fruits. Heatmaps show the fold change in expression of genes associated with ethylene signal transduction (**d**) and cell wall metabolism (**e**, **f**)**.** The transcript levels of *ACO1* (**g**), *ACO5* (**h**), *ERF4* (**i**), *Pti5* (**j**), *AP2a* (**k**), *AP2c* (**l**) *TBG7* (**m**) and *XTH9* (**n**) were analysed by qRT–PCR. In (**g**) to (**n**), each data represents a mean of three replicates.

To explore the *VAHOX1*-mediated regulation of fruit ripening at the molecular level, a transcriptome analysis of the WT and *VAHOX1*-RNAi fruits was performed. Relative to the WT, 1640 upregulated and 649 downregulated genes were identified in the *VAHOX1*-RNAi fruits (Fig. 6a). Kyoto Encyclopedia of Genes and Genomes (KEGG) pathway enrichment and Gene Ontology (GO) enrichment analyses indicated that the silencing of *VAHOX1* affected a variety of metabolic processes, including plant hormone signal transduction, biosynthesis of secondary metabolites, flavonoid biosynthesis, and carotenoid biosynthesis (Fig. 6b and c). Given the characteristics of *VAHOX1* and the phenotypes of transgenic tomato plants regarding fruit ripening, we focused on genes involved in ethylene and ripening-related processes. DEG functional annotation revealed that a large set of genes were associated with ethylene synthesis and signal transduction and with cell wall degradation. Heatmaps were generated to better visualize the differences in ripening-related genes (Fig. 6d–f). The transcripts of ethylene biosynthetic genes, such as *ACO1* and *ACO5* were increased in the *VAHOX1*-RNAi fruits. Ethylene-responsive genes, such as *ERF4*/*ERF.B3*, *AP2a*, and *AP2c* were repressed in the *VAHOX1*-RNAi fruits, whereas *Pti5*/*ERF.C6* was induced. Transcript levels of cell wall catabolism-related proteins, such as β-galactosidase precursor (TBG), endo-β-1,4-glucanases (Cels), expansin (EXP), xyloglucan endotransglucosylase hydrolase (XTH) and pectin methylesterase (PME) were altered. Furthermore, a total of eight genes (*ACO1*, *ACO5*, *ERF4*/*ERF.B3*, *Pti5*/*ERF.C6*, *XTH9*, *TBG7*, *AP2a*, and *AP2c*) were subjected to qRT–PCR analysis in B + 4 fruits. The expression results were in line with the RNA-seq data (Fig. 6g–n). These data suggest that VAHOX1 affects fruit ripening by modulating ripening-related gene expression.

### VAHOX1 can activate the promoter activity of *AP2a*

**Figure 7:**
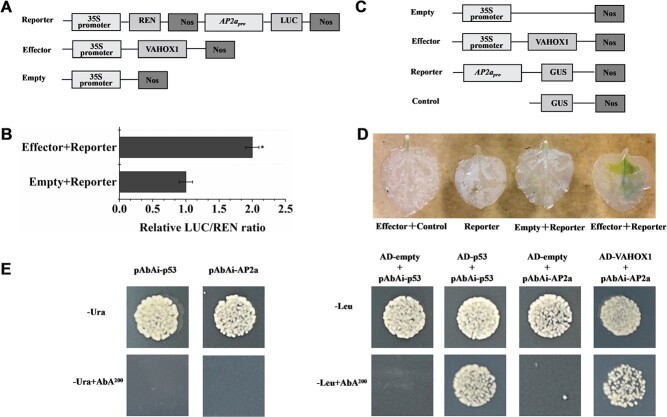
VAHOX1 activates the transcription of *AP2a*. **a** and **c** Schematic diagrams of the effector and reporter constructs used in the transient expression. Dual-luciferase assay (**b**) and histochemical assay (**d**) for VAHOX1 activating *AP2a* promoter activity. Values represent the means of three replicate. **e** Y1H assay showed VAHOX1 could activate the *AP2a* promoter in yeast. Negative control, pAbAi-p53 + AD-empty. Positive control, pAbAi-p53 + AD-p53.

Given that the artificial suppression or enhancement of *VAHOX1* in tomato altered fruit ripening and softening and affected the transcription of genes associated with ethylene signal transduction and cell wall metabolism, we checked whether VAHOX1 directly regulate the transcription of the genes mentioned in Fig. 6 through a dual-luciferase assay. The effector vector harbour *VAHOX1*; the double-reporter vectors contained the promoters of genes (*ACO1*, *ACO5*, *ERF4*, *Pti5*, *XTH9*, *TBG7*, *AP2a*, and *AP2c*), respectively. Interestingly, the dual-luciferase experiments revealed that VAHOX1 only distinctly promoted the promoter activity of *AP2a in vivo* (Fig. 7a and b). The relative LUC/REN ratios in tobacco leaves cotransformed with the effector vector harbouring *VAHOX1* and the other double-reporter plasmids showed no notable differences (data not shown). Meanwhile, cotransformation with the effector vector harbouring *VAHOX1* remarkably activated the GUS reporter gene driven by the *AP2a* promoter in tobacco leaves (Fig. 7c and d). Additionally, a Y1H assay confirmed the interaction of VAHOX1 with the promoter fragment of *AP2a***(**Fig. 7e**)**. Our results indicate that VAHOX1 regulates fruit ripening by activating the transcription of *AP2a.*

## Discussion

### HD-zip family transcription factor VAHOX1 affects tomato fruit ripening

In addition to studies highlighting the roles of HD-Zip I proteins in regulating environmental stress responses [51, 52], anthocyanin biosynthesis [53], and both leaf and flower senescence [54, 55], there have also been many reports describing the importance of these subfamily proteins in mediating ethylene signalling and fruit ripening [33]. For instance, HaHB4 negatively regulates sunflower senescence by reducing ethylene synthesis and inhibiting its signal transduction [56]. Recently, a study found that four banana HD-Zip proteins may participate in fruit ripening by stimulating gene expression associated with ethylene synthesis and cell wall degradation [57]. The tomato HD-Zip I TF LeHB-1 regulates fruit ripening by controlling *ACO1* mRNA accumulation [33]. However, despite these observations, no other HD-Zip proteins related to fruit ripening have been reported in tomato. Here, the effect of HD-Zip transcription factor VAHOX1 on tomato fruit ripening was investigated. We found that fruit ripening was accelerated in the *VAHOX1*-RNAi lines but delayed in the *VAHOX1*-OE plants (Fig. 3). Our study indicates that VAHOX1 has a regulatory function of delaying tomato fruit ripening. Because most of the identified TFs function to accelerate fruit ripening [10, 58], this work has advanced the understanding of the transcriptional regulation of fleshy fruit ripening.

### VAHOX1 influences ethylene production and ethylene sensitivity

**Figure 8:**
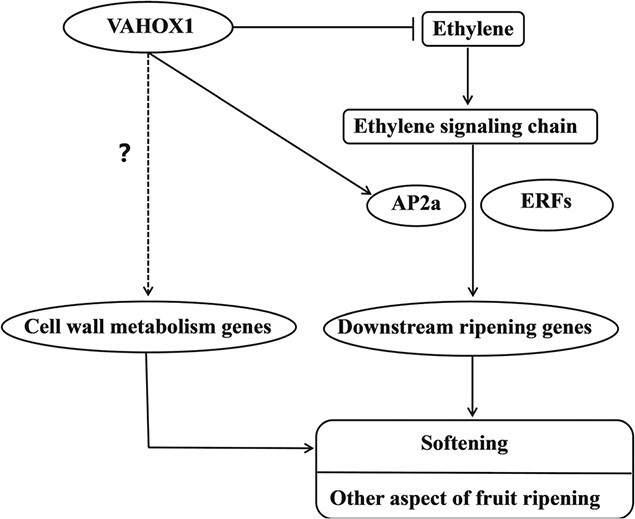
Proposed model depicting the role of VAHOX1 in tomato fruit ripening.

Climacteric fruits such as tomato display a dramatic increase in ethylene and respiration during ripening initiation [59]. In this study, we found that *VAHOX1* was expressed more highly in the B + 4 fruits of wild-type tomato plants (Fig. 2a), and its expression could be induced by ACC or ethylene but repressed by 1-MCP (Fig. 2c and d). Silencing of *VAHOX1* resulted in increased ethylene production, whereas *VAHOX1*-overexpressing fruits exhibited slightly lower ethylene production (Fig. 3d). This result implies that VAHOX1 may be a negative regulator of ethylene production (Fig. 8). The expression of genes correlated with ethylene biosynthesis were changed in the transgenic plants (Fig. 6d, g and h). Relative to the WT, the *VAHOX1-*RNAi fruits displayed enhanced transcription of two ethylene biosynthesis genes, *ACO1* and *ACO5* (Fig. 6g and h). The inhibition of *ACO1* results in reduced ethylene biosynthesis and delayed onset of ripening in tomato fruits [60–62]. Nevertheless, dual-luciferase experiments showed that VAHOX1 could not promote the promoter activities of *ACO1* and *ACO5* in tobacco leaves. Tomato fruit ripening is a programmed process mediated by complex transcriptional regulatory networks, and whether VAHOX1 directly regulates other ethylene biosynthesis genes needs to be further studied.

Ethylene treatment experiments showed that *VAHOX1* could affect the sensitivity of tomato to ethylene (Fig. 4a and j). Moreover, the artificial suppression or enhancement of *VAHOX1* in tomato altered the expression of ethylene-responsive genes (Fig. 6d, i–l). Tomato plants with upregulated mRNA levels of the ethylene response factor *Pti4/5/6* exhibited accelerated fruit ripening and enhanced disease resistance [63]. Ethylene response factor *ERF.B3*/*ERF4* has a considerable role in fruit development and ripening. Overexpression of *ERF.B3-SRDX* causes delayed fruit ripening and reduced pigment accumulation [64]. In this work, we found that the transcript levels of *ERF.B3*/*ERF4* were decreased in the RNAi fruits but elevated in the OE fruits, while *Pti5*/*ERF.C6* showed the opposite expression (Fig. 6i and j). The expression of the tomato APETALA2/ERF gene *AP2a* is increased during fruit ripening and stimulated by ethylene. Repression of *AP2a* in tomato results in increased ethylene production and early fruit senescence [21, 22]. In our experiment, *VAHOX1*-silenced and *VAHOX1*-OE fruits exhibited noticeably reduced and increased *AP2a* expression, respectively (Fig. 6k). Moreover, *VAHOX1*-RNAi fruits exhibited a higher physiological water loss rate compared with WT fruits (Fig. 5d). The Y1H assay, dual-luciferase transient expression assay and the histochemical analysis of GUS activity showed that VAHOX1 directly activates the transcription of *AP2a* (Fig. 7e). Therefore, our results suggest that VAHOX1 regulates ethylene-dependent fruit ripening, and VAHOX1 negatively regulates fruit ripening at least in part by altering the expression of *AP2a* (Fig. 8).

### VAHOX1 affects cell wall metabolism and may participate in the responses of tomato fruits to biotic stresses

Many HD-Zip TFs have been reported to control cell wall development and modification. Four *MaHDZs* can activate the transcription of cell wall modification-related genes in banana [57]. Arabidopsis class III HD–ZIP gene *AtHB15* is important for pith secondary wall development [65]. The maize OCL4 affects the division and/or differentiation of the anther cell wall [66]. In addition, ethylene has been considered a key factor in cell wall metabolism. For instance, the ethephon and 1-MCP treatments of strawberry fruits lead to altered expression of genes involved in cell wall metabolism and cellulose and pectin contents [67]. In this work, the disruption of the normal expression of *VAHOX1* resulted in increased ethylene production and altered expression of a large number of genes related to cell wall metabolism in tomato fruits. Thus, it is easy to understand the short shelf life of *VAHOX1-*RNAi fruits (Fig. 5). Nevertheless, dual-luciferase experiments indicated that VAHOX1 could not activate the transcription of the cell wall metabolism-related genes *TBG7* and *XTH9* in tobacco leaves. VAHOX1 may indirectly regulate cell wall-related genes during tomato fruit ripening, or VAHOX1 may directly regulate other cell wall modification-related genes, which needs further investigation (Fig. 8).

HD-Zip TFs are involved in the developmental processes associated with changes in environmental conditions [68]. For example, Arabidopsis plants ectopically expressing maize *Zmhdz10* displayed enhanced salt and drought resistance [69]. The upregulation of *HAHB4* increases jasmonic acid levels and improves susceptibility to pathogen infections in Arabidopsis [70]. Here, the analysis of DEGs suggested that the plant–pathogen interaction was the most enriched KEGG pathway (Fig. 6b). Fruit losses caused by pathogen infections during the postharvest storage and handling of fruits have led to enormous economic losses around the world [71]. Our current work may spur more interest in further studying the involvement of VAHOX1 in the response of fruits to pathogen infection.

Taken together, our work demonstrates that VAHOX1 functions in fruit ripening as an inhibitor. These results expand our knowledge about the physiological significance of HD-Zip TFs in plant growth and development and further perfect the transcriptional regulatory network of tomato fruit ripening.

## Materials and methods

### Plant materials and culture conditions

Tomato (*Solanum lycopersicum* cv. Ailsa Craig), *Nr* and *rin* plants were grown under glasshouse conditions as described in our previous report [34]. Flowers were tagged at the stage of anthesis to assess fruit ripening stages. Fruits were harvested at these stages: immature green fruit (IMG), mature green fruit (MG), breaker stage fruit (B), and 4 and 7 days after breaker stage (B + 4 and B + 7, respectively).

### Plasmids construction and plant transformation

To generate the *VAHOX1* overexpression vector, the CDS of *VAHOX1* (GenBank Accession No. NM_001247321) was cloned into the overexpression vector pBI121. A 360-bp specific fragment was cloned and inserted into the RNA interference vector pBIN19 for the *VAHOX1* RNAi construct. The resulting vectors were introduced into tomato through transformation mediated by *Agrobacterium tumefaciens* (strain LBA4404) as described elsewhere [35]. NPTII-F/R primers were used to detect positive transgenic plants by PCR amplification. All primers used in this study are listed in Table S1 (see online supplementary material).

### Sequence analyses

Multiple sequence alignment of tomato VAHOX1 and other HD-Zip I members from different plant species was performed by DNAMAN (v. 6.0) software. Structural domains were analysed using Scan Prosite (http://prosite.expasy.org/scanprosite/). The Neighbor-joining (NJ) phylogenetic tree was generated with MEGA version 6. Sequences included in the phylogenetic tree are as follows: AtHB1 (AAF01532), LeHB-1 (CAP16664), MdHB1 (ASR12269), GmHDZ20 (XP_003524980), VAHOX1 (NP_001234250), AtHB13 (NP_177136), H52 (NP_001234571), AtHB6 (NP_565536), AtHB16 (NP_195716), OsHox22 (XP_015634133), PuHox52 (QEQ92614), RhHB1 (AIN45171), AtHB7 (AY091364), AtHB12 (BT002206), HaHB4 (AAA63768).

### Subcellular localization and transactivation assay

For generating *35S::VAHOX1*-*GFP* recombinant construct, the CDS of *VAHOX1* was cloned into the pBI121 vector. *35S::VAHOX1*-*GFP* and *35S::GFP* were transferred to *A. tumefaciens* strain GV3101 and then injected into *Nicotiana benthamiana* leaves, respectively. The fluorescence images of localization samples were acquired on a confocal laser scanning microscope (Leica TCS SP8) after 72 h of infiltration, HY5-RFP was used as a nuclear location maker.

The full-length and truncated forms of *VAHOX1* were cloned into pGBKT7 (BD) vector. The different recombinant constructs were introduced into yeast strain Y2H-Gold. SD/−Trp-His-Ade and SD/−Trp-His-Ade/+X-α-Gal mediums were used to evaluate the transcriptional activation activity based on the growth status of transformed yeast cells.

### Treatments of tomato fruits with different hormones

The wild-type (WT) MG fruits were harvested and respectively injected with 100 μM 1-aminocyclopropane-1-carboxylic acid (ACC), indole-3-acetic acid (IAA) and abscisic acid (ABA) solution (pH 5.6) containing 10 mM MES and 3% sorbitol as described [36, 37]. After treatments, fruits were incubated in a controlled growth chamber for 4 days [34]. Pericarps were collected to conduct the quantitative real-time polymerase chain reaction (qRT–PCR) assay.

For ethylene and methylcyclopropen (1-MCP) treatments, MG fruits were treated with 1 mg/L 1-MCP, 10 ppm ethephon (Eth) solution or distilled water in an incubator for 24 h and then transferred to open air for 4 days. The pericarps of fruits treated for 2 days were collected for gene expression analysis by qRT–PCR.

### Ethylene measurement and ethylene triple response assay

Fruits were harvested at the B + 4 stage and placed in open 240 mL bottles for 3 h. These fruits were then enclosed in glass jars at 25°C for 3 h. Gas chromatography (GC) was used to measure 1 mL of gas sample as previously described [21]. The measurement was performed with at least six tomato fruits for each line.

Ethylene triple-response assay was conducted as described elsewhere [38]. For ethylene response testing, surface sterilized seeds of the wild-type plants were sown on MS medium containing different concentrations of ACC, respectively. For phenotypic observation, sterilized seeds of the control and transgenic plants were sown on MS medium supplemented with 0, 5.0, and 10.0 μM ACC, respectively. Hypocotyl and root elongation of seedlings were measured after 6 days of incubation in the dark. Meanwhile, the expression of *VAHOX1* was also analysed. For each line, at least 16 seedlings were counted.

### Measurements of carotenoid, lycopene, and pectin content

Carotenoid was extracted and calculated following the methods reported previously [39]. Lycopene content was measured as described [40]. Pectin content was determined using a kit (Comin Suzhou, China) following the recommended protocol.

### Water loss

Tomato fruits were picked at the B stage and placed in a greenhouse under controlled conditions (25–26°C and 80% relative humidity). The fruits were weighed every 5 days until 20 days. The water loss rate was calculated according to the equation listed in the previous report [41].

### Microscopic observations

The fresh pericarp sections of fruits stored for 20 days were prepared and fixed in FAA liquid (50% ethanol: acetic acid: formaldehyde, 18:1:1 v/v) to examine the cell wall structure of pericarp. The fixed tomato fruit pericarp was dehydrated with a series of graded ethanol, then fixed, sliced, dewaxed, and stained. All sections were observed using an optical microscope (Nikon E100).

### RNA-sequencing

RNA-sequencing (RNA-seq) analysis was performed on fruits of the *VAHOX1*-RNAi5 and the wild-type plants at the B + 4 stage. The specific operation steps are based on our previous report [34].

### Transient expression assays

The coding sequence of VAHOX1 was cloned into the pGreenII 62-SK vector as effector, while the promoter fragment of *AP2a* was inserted into the pGreenII 0800-LUC and pGreenII 0800-GUS vector as reporters, respectively [42, 43]. The firefly luciferase (LUC) and Renilla luciferase (REN) activities were measured based on the description of previous report [38]. GUS histochemical experiment was performed according to the described method [38, 44].

### Y1H assay

A Y1H experiment was conducted using the Matchmaker Gold Y1H System. The promoter fragment of *AP2a* was ligated into the pAbAi vector to produce a bait vector, which was transformed into yeast strain Y1H-Gold. To avoid self-activation, the minimal inhibitory concentration of aureobasidin A (AbA) was screened. The CDS of *VAHOX1* was ligated into pGADT7 as a prey construct, which was transformed into the bait yeast strain and grown on SD-Leu/AbA medium.

### Statistical analysis

All data are means ± standard deviation of three independent experiments. Pairwise comparison was assessed by Student’s *t*-test (^*^P < 0.05).

## Acknowledgments

The project was supported by the National Natural Science Foundation of China (no. 31872121) and the Natural Science Foundation of Chongqing, China (csts2019jcyj-msxmX0094, cstc2019jcyj-msxmX0361).

## Author contributions

Z.H. and X.C. designed experiments; F.L., M.F., S.Z., Q.X., and G.C. conducted the experiments; S.Z. and F.L. analysed the data and F.L. wrote the manuscript.

## Data availability

All relevant data are included within the article and its supplemental files.

## Conflict of interest

The authors declare no competing interests.

## Supplementary data

Supplementary data is available at *Horticulture Research* online.

## Supplementary Material

Web_Material_uhac236
