# Supplementary material for: A tomato HD-zip I transcription factor, VAHOX1, acts as a negative regulator of fruit ripening
Source: Hortic Res. 2022 Oct 19;10(1):uhac236. doi: 10.1093/hr/uhac236 (PMC9832867; doi:10.1093/hr/uhac236)
Supplement: Web_Material_uhac236 [file web_material_uhac236.zip › Supplementary figures.docx]

**Supplementary Fig. S1**


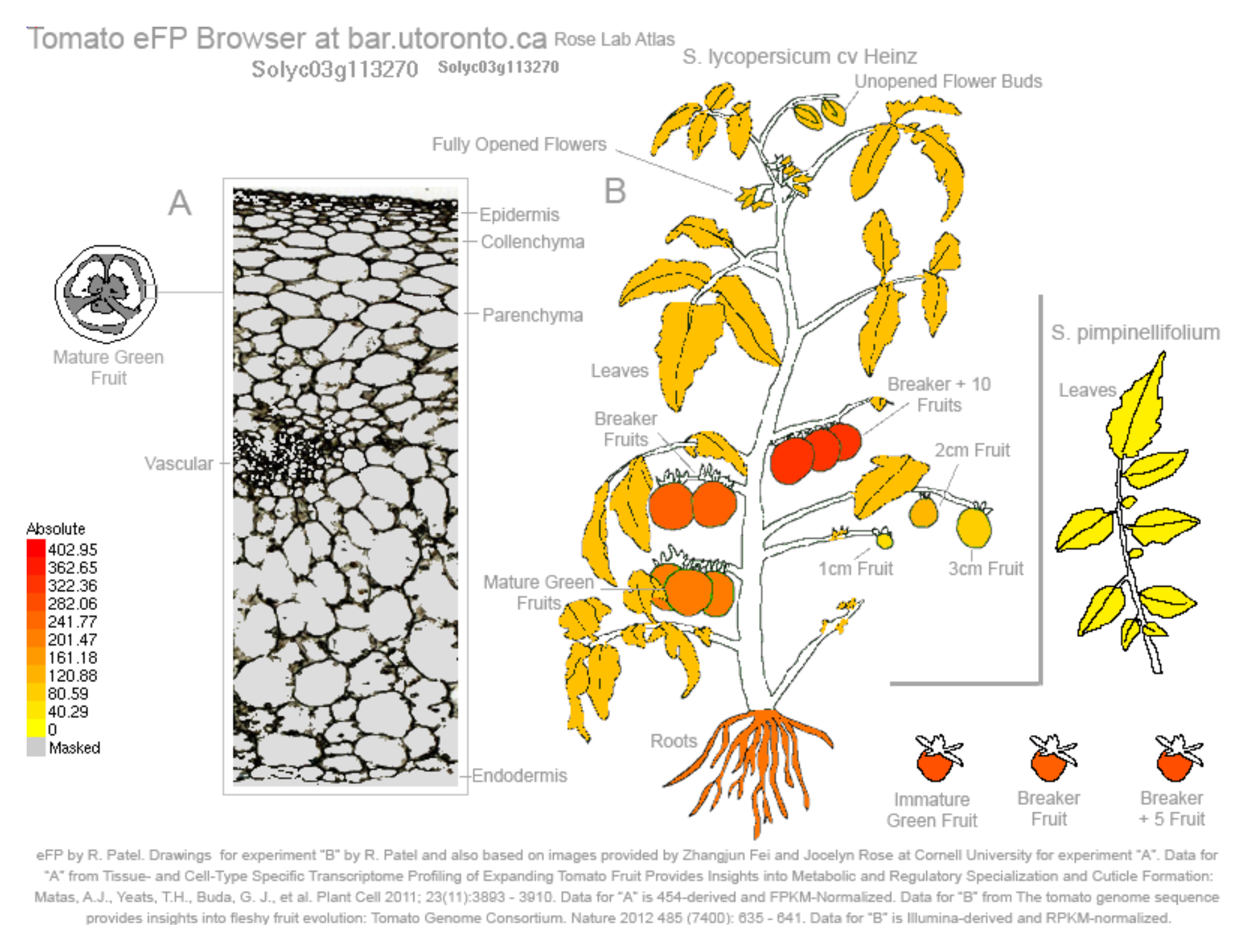


**Supplementary Fig. S1.** The expression profile of *VAHOX1* in various tissues from the online Tomato eFP Browse.

**Supplementary Fig. S2**


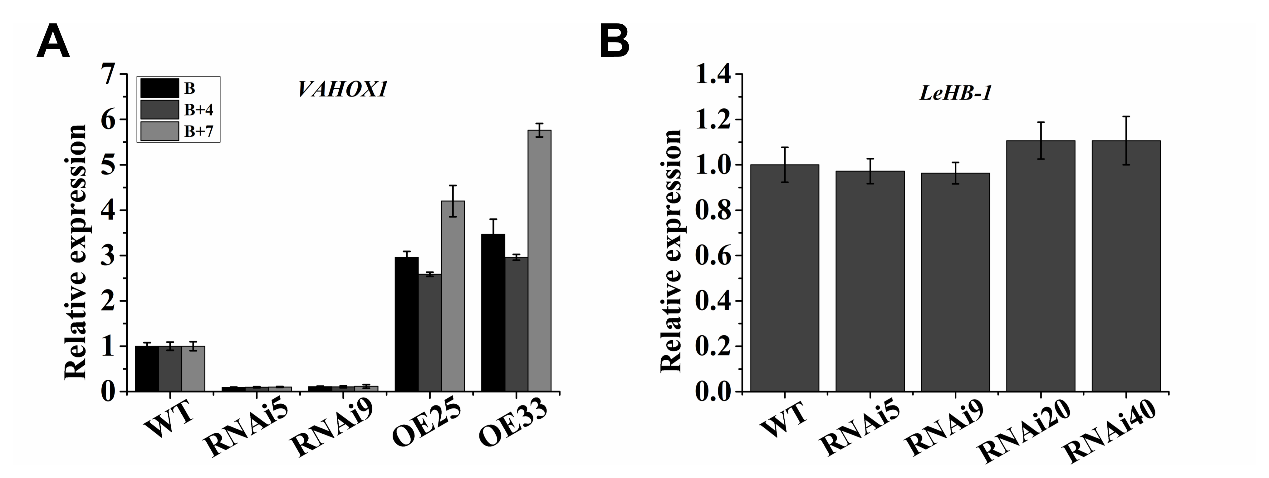


**Supplementary Fig. S2.** Relative transcript levels of *VAHOX1* in the *VAHOX1*-RNAi, *VAHOX1*-OE and wild-type (WT) fruits at different stages (**A**), and the relative transcript levels of *LeHB-1* in the *VAHOX1*-RNAi and WT fruits at the B stage (**B**). Values are means ± standard error (SE) of three biological replicates. Asterisks indicate significant differences (*P* < 0.05). The same as below.

**Supplementary Fig. S3**


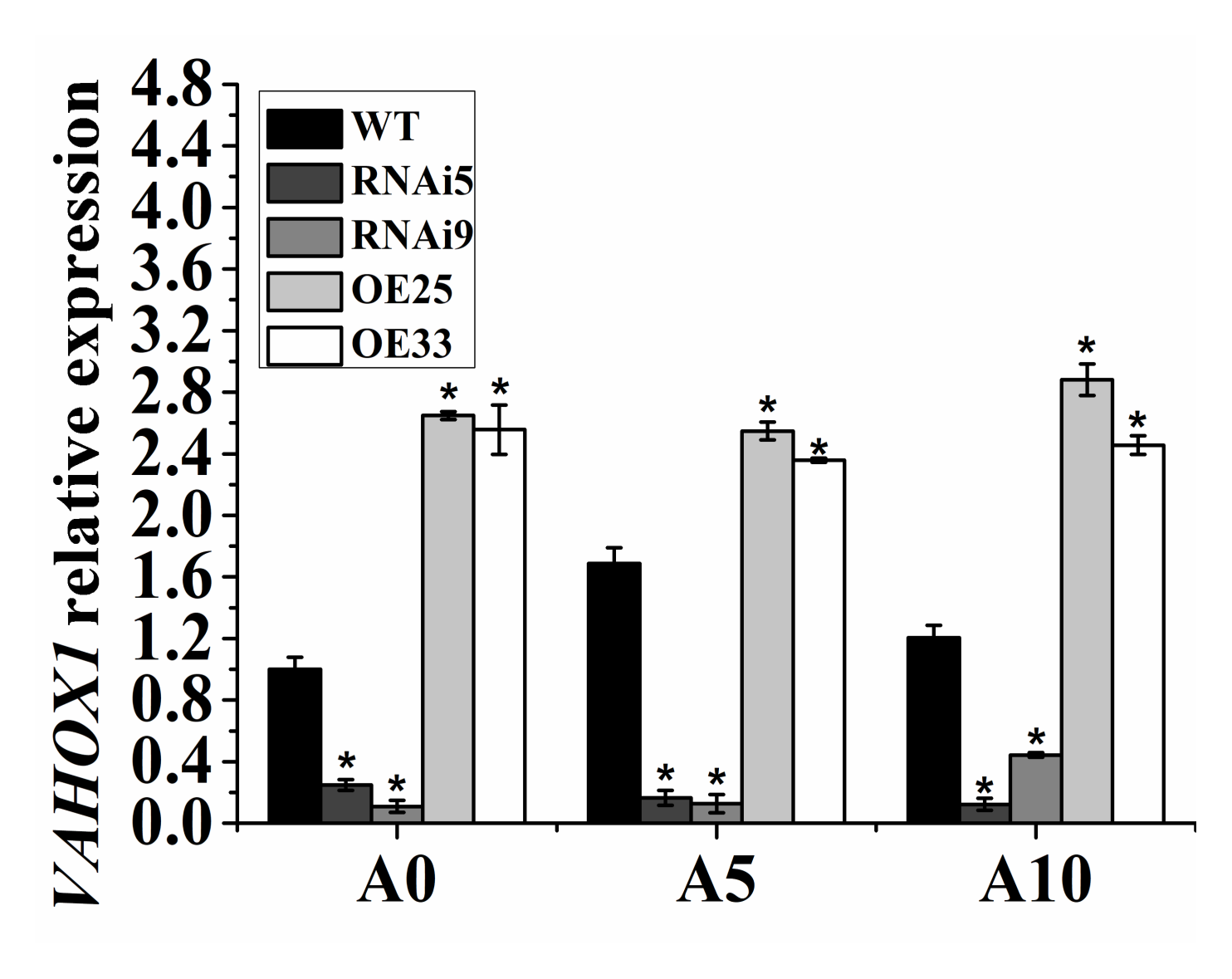


**Supplementary Fig. S3.** *VAHOX1* expression in the *VAHOX1*-RNAi, *VAHOX1-*OE and WT seedlings under air (A0), 5 μM ACC (A5) and 10 μM ACC (A10), respectively. Values are means ± SE of three biological replicates.

**Supplementary Fig. S4**


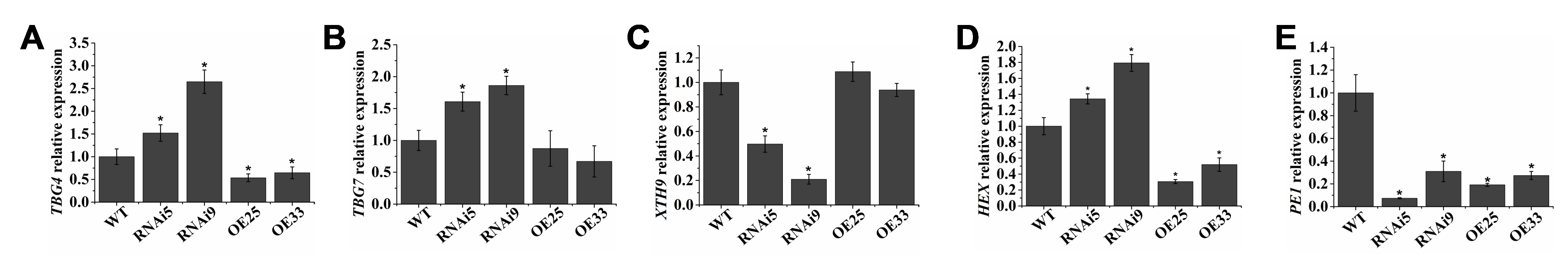


**Supplementary Fig. S4.** (a–e) Relative expression of cell wall metabolism genes in the WT, *VAHOX1*-RNAi and *VAHOX1*-OE fruits. Values are means ± SE of three biological replicates.
