# Supplementary material for: A tomato HD-zip I transcription factor, VAHOX1, acts as a negative regulator of fruit ripening
Source: Hortic Res. 2022 Oct 19;10(1):uhac236. doi: 10.1093/hr/uhac236 (PMC9832867; doi:10.1093/hr/uhac236)
Supplement: Web_Material_uhac236 [file web_material_uhac236.zip › Supplementary table.docx]

**Supplementary Table S1 All the primer sequences used in this study.**

| **Primer name** | **Forward primer Sequence (5' →3')** | **Reverse primer Sequence (5' →3')** | **Experiments** |
| --- | --- | --- | --- |
| **Primers for constructs in plant transformation** | | | |
| *VAHOX1*-OE | GCTCTAGAATTACATAACGAAACGAGCGGAT | CGAGCTCTGTGCTTTCTATACAATGTCTCC | To establish *VAHOX1* overexpression lines |
| *VAHOX1*-RNAi | GGGGTACCAAGCTTCGAAACGAGCGGATATGGGT | CCGCTCGAGTCTAGAGCCTCTTCTTCTCCGCTTGA | To establish *VAHOX1* RNAi lines |
| *VAHOX1*-GFP | GCTCTAGAATGATGGCTCCAGGGATTCTCTA | CGGGATCCATAAGTCCAGAAACCAAAACCTTG | GFP, transient expression |
| *VAHOX1*-62-SK | GCTCTAGAATGATGGCTCCAGGGATTCTCTA | GGGGTACCTTAATAAGTCCAGAAACCAAAACCT | To establish effector, transient expression |
| *pACO1*-800 | GGGGTACCGCTTATTTACGGTCCTAACAGAGTT | CCGCTCGAGAATGAATAATTATTATATTCCAAGTCTTTA | To establish reporter, transient expression |
| *pACO5*-800 | GGGGTACCTAATCCAAGTTACACATTGCCAAAA | CCGCTCGAGAGGAAGGTACTTGGCCCGACTA | To establish reporter, transient expression |
| *pERF4*-800 | GGGGTACCCTACATCACACTCCCATCCTTCTTA | CCGCTCGAGGCAGTATTTTGAGAATTGCAGTGAA | To establish reporter, transient expression |
| *pPti5*-800 | TGCTTTGCATACAATTAAATCGAAG | AGCTTTTATTAGAGGGGATTTTGGT | To establish reporter, transient expression |
| *pTBG7*-800 | GGGGTACCACTTCACCACTGATTATTTTGCTTC | CCGCTCGAGATTCCGACGAGAGTTGGCATT | To establish reporter, transient expression |
| *pXTH9*-800 | GGGGTACCGGATGATTGAGATATCTTCACTTTT | CCGCTCGAGATTTTGATGTTATTGGTTTGGTTA | To establish reporter, transient expression |
| *pAP2a*-800 | GGGGTACCAAAAGAGGAAAAAAGGTGGGTCC | CCGCTCGAGGTGTATTTTTCGATATTTGGTTGC | To establish reporter, transient expression |
| *pAP2c*-800 | GGGGTACCGAAATAGCGGCAGAAAGTGGC | CCGCTCGAGTCTCTACAAAAAATACCCAATCCAA | To establish reporter, transient expression |
| **Primers for transgenic plants identification** | | | |
| *NPT II* | CTCAGAAGAACTCGTCAAGAAGG | GACTGGGCACAACAGACAATC | Positive transgenic plants detection |
| **Primers for constructs in yeast cell** | | | |
| *VAHOX1* (1-324aa) | GGAATTCCATATGATGATGGCTCCAGGGATTCTCTA | CCGGAATTCTTAATAAGTCCAGAAACCAAAACCTT | Transactivation activity assay |
| *VAHOX1* (1-188aa) | GGAATTCCATATGATGATGGCTCCAGGGATTCTCTA | CCGGAATTCAAACAGCTTACCGGTGAGACGG | Transactivation activity assay |
| *VAHOX1* (1-145aa) | GGAATTCCATATGATGATGGCTCCAGGGATTCTCTA | CCGGAATTCAGTCTTCCATCGCGCACGAC | Transactivation activity assay |
| *VAHOX1* (1-88aa) | GGAATTCCATATGATGATGGCTCCAGGGATTCTCTA | CCGGAATTCTTGATGAAGATATTCCCCCAATTC | Transactivation activity assay |
| *VAHOX1* (89-324aa) | GGAATTCCATATGGCGGAGAAGAAGAGGCGACTTAC | CCGGAATTCTTAATAAGTCCAGAAACCAAAACCTT | Transactivation activity assay |
| *VAHOX1* (89-188aa) | GGAATTCCATATGGCGGAGAAGAAGAGGCGACTTAC | CCGGAATTCAAACAGCTTACCGGTGAGACGG | Transactivation activity assay |
| *VAHOX1* (89-145aa) | GGAATTCCATATGGCGGAGAAGAAGAGGCGACTTAC | CCGGAATTCAGTCTTCCATCGCGCACGAC | Transactivation activity assay |
| *VAHOX1* (146-324aa) | GGAATTCCATATGAAGCAGCTCGAGAAAGATTATGATG | CCGGAATTCTTAATAAGTCCAGAAACCAAAACCTT | Transactivation activity assay |
| *VAHOX1* (146-188aa) | GGAATTCCATATGAAGCAGCTCGAGAAAGATTATGATG | CCGGAATTCAAACAGCTTACCGGTGAGACGG | Transactivation activity assay |
| *VAHOX1* (189-324aa) | GGAATTCCATATGATCAAAGAGAAAGGAAATGGGC | CCGGAATTCTTAATAAGTCCAGAAACCAAAACCTT | Transactivation activity assay |
| *pAP2a*-Y1H | GGGGTACCTGAAAGTTGATATTCTTTTTTATAAGGA | CCGCTCGAGGTTCGATTTTTTTTTGAATTATTGAGT | Yeast one-hybrid (Y1H) assay |
| **Primers for qRT-PCR** | | | |
| q*SlCAC* | CCTCCGTTGTGATGTAACTGG | ATTGGTGGAAAGTAACATCATCG | qRT-PCR |
| q*VAHOX1* | CTGAAGTTTTCCGTCTCACCG | GTGTTTGTGTTCATCGCGCA | qRT-PCR |
| q*ACO1* | ACAAACAGACGGGACACGAA | CTCTTTGGCTTGAAACTTGA | qRT-PCR |
| q*ACO5* | CATCAGCAACACAGACTGGGAAA | TTCTGAAAGATTTTCTGCAAGTTTG | qRT-PCR |
| q*ERF4* | CGGAGATAAGAGATCCAAGTCGAA | CTTAAACGCTGCACAATCATAAGC | qRT-PCR |
| q*Pti5* | TTAGCTTATGATAGAGCGGCTTTTA | TTCGTAGTGTAACTATTTGAGCACA | qRT-PCR |
| q*TBG4* | AAATGGTGAAGGCGTAGGTCG | AGGTTGTCCGCAGTTAGTCTGG | qRT-PCR |
| q*TBG7* | TTTGGAACTCCTATTGCTCTAAAGG | CGCAGTCAAGTCCATAGTCCCAG | qRT-PCR |
| q*XTH9* | GTCTGGAATCCTCATCGCATAGT | CACTCTGCCTCCTTGGGTTG | qRT-PCR |
| q*AP2a* | GAATGTACTGATAATGCAACGGACC | GCTGCTCGGAGTCTGAACCTTA | qRT-PCR |
| q*AP2c* | TCCAGCCTCCTTGAAGCATAAC | TTCCACCATTGCTGCCACTT | qRT-PCR |
| q*HEX* | GCAGAAGCATTGTGGTCAGGA | TCAGCACCTATTCCCCTAGAAAC | qRT-PCR |
| q*PE1* | GCTTGCGTCTTTGACAACTCAGG | GTGCCACCACTGCATTCGCTAT | qRT-PCR |
| q*HB-1* | TCTCTGATTTTGACTCCGTTCG | TTTCATTTCCCCCTGCTCCT | qRT-PCR |
